# RUFY3 Predicts Poor Prognosis and Promotes Metastasis through Epithelial-mesenchymal Transition in Lung Adenocarcinoma

**DOI:** 10.7150/jca.35072

**Published:** 2019-10-17

**Authors:** Wanfu Men, Wenya Li, Yu Li, Jungang Zhao, Xiaohan Qu, Peiwen Li, Shulei Gong

**Affiliations:** 1 Department of Thoracic Surgery, The First Affiliated Hospital of China Medical University, Shenyang, Liaoning 110001, P.R. China; 2 Department of Thoracic Surgery, Shenjing Affiliated Hospital of China Medical University, Shenyang, Liaoning 110004, P.R. China

**Keywords:** RUFY3 (RUN and FYVE domain-containing protein 3), lung adenocarcinoma, prognosis, EMT

## Abstract

**Background**: RUFY3 (RUN and FYVE domain-containing protein 3) has been shown to participate in cell migration, membrane transportation, and cellular signaling and is dysregulated in several cancer processes. However, the role of RUFY3 in lung cancer remains unclear. In the present study, we aimed to study the expression of RUFY3 and assess its clinical significance in lung adenocarcinoma.

**Materials and Methods**: We used immunohistochemistry to detect RUFY3 protein expression in human lung adenocarcinoma and adjacent normal lung tissue from 125 patients who underwent surgical resection of the lung cancer. RUFY3 expression was assessed in association with clinicopathological characteristics and clinical prognosis of lung adenocarcinoma patients. The expression of RUFY3 in three different lung adenocarcinoma cell lines and one normal lung epithelial cell (BEAS-2B) was detected by western blot. RNAi technique was used to silence RUFY3. We assessed cell migration by Trans-well assay and wound healing assay.

**Results**: In lung adenocarcinoma tissues, RUFY3 protein was significantly upregulated compared to paired normal lung tissues. High cytoplasmic RUFY3 levels were associated with lymph node metastasis, TNM staging, and survival status. Patients with the highest expression level of RUFY3 had a shorter survival time than patients with the lowest expression. Inhibition of RUFY3 by siRNA inhibited cell migration. Furthermore, silence of RUFY3 lead to up-regulation of E-cadherin, but down-regulation of N-cadherin, Vimentin and Slug.

**Conclusions**: Our study is first to demonstrated that abnormal expression of RUFY3 indicates poor prognosis in lung adenocarcinoma and also indicates that RUFY3 may be related to EMT process. This highlights the potential of RUFY3 as a novel prognostic biomarker for lung adenocarcinoma.

## Introduction

Lung cancer is considered one of several cancers with the highest incidence and fatality rate worldwide 1, 2. In recent years, adenocarcinoma is gradually becoming a major component of lung cancer and indicates poor prognosis, especially in China 3. However, as variable gene mutations of lung adenocarcinoma are not well investigated 4, no satisfactory treatment is available. Therefore, identifying potential effective therapeutic molecules is significant in cancer treatment.

RUFY3 (RUN and FYVE domain-containing protein 3) protein belongs to the RUN and FYVE domain protein families, the feature of which contains a RUN structure in the amino end and a FYVE domain structure in the carboxyl end^5^, ^6^. Sequencing and genome analysis demonstrated that the RUFY family consists of four protein members: RUFY1/rabip4, RUFY2, RUFY3/singar, and RUFY4 7-10. RUFY proteins are located primarily in the early endosomes of cells, and participate in several cellular processes, including cell migration, cytoskeletal dynamics 11, lipid modification 12, membrane transport 13, and cellular signaling 14.

RUFY3 is also known as RIPX and Singar1, and contains 469 amino acids widely distributed in the neurons of the hippocampus and can enrich cone and axon growth. RUFY3 has common characteristics of other members in the RUFY family containing a RUN domain that plays an important role in the Ras sample GTP signaling pathway. It has so far been demonstrated that RUFY3 is similar to its homologues, such as RPIP8 15 and RPIP9 16, can interact with activated Rab 17, and may also be associated with Rap2 in the GTP enzyme dependent pattern. Recently one research showed that in gastric cancer, RUFY3 can interact with activated P21 kinase-1 (PAK1) and promote gastric cancer metastasis 18. Another research showed that RUFY3 can interact with FOXK1 and promotes invasion and metastasis in colorectal cancer 19. These results demonstrated that RUFY3 may play a role in promoting the development of cancer and targeting it may provide a breakthrough in cancer treatment. However, the role of RUFY3 in lung cancer remains unclear.

## Materials and Methods

### Patient and tissue samples

In this study, we selected a total of 125 formalin-fixed and paraffin-embedded lung adenocarcinoma samples and their corresponding normal samples. All cancer patients were from the Department of Thoracic Surgery and underwent lung cancer resection with standard lymph node dissection at the First Hospital of China Medical University between 2010 and 2012. Prior to this study, the patients and their families had been informed and informed consent was signed. Inclusion criteria: postoperative pathological diagnosis was primary lung adenocarcinoma. Exclusion criteria: pulmonary metastatic carcinoma, anesthesia intolerance, severe cardiopulmonary dysfunction, preoperative radiotherapy or chemotherapy. We collected pathological characteristics of postoperative patients, including age, sex, smoking, tumor size, differentiation, lymph node metastasis, and TNM staging. The TNM stages are staged according to UICC (Union for International Cancer Control) version 8 (2017). All patients were followed up by telephone with complete follow-up data.

### Immunohistochemistry

All tissues were fixed by formalin and embedded with paraffin and sliced into 4 μm thick slices. Immunohistochemical staining was conducted using Histostain-Plus kit (Zymed Laboratories Inc., San Francisco, CA, USA) with diaminobenzidine (DAB) as the chromogen. The section was redyed with hematin. Negative control group was used for the non-immunological rabbit serum and PBS for primary antibodies.

### Evaluation of immunohistochemical staining results

Immunohistochemical staining evaluation was performed by two experienced clinical pathologists independently. Immunohistochemical scoring system was semi-quantitative. Staining intensity was scored 0-3 points (0, no staining; 1, weak staining; 2, moderate staining; and 3, strong staining). The cell percentage was scored 0-3 points (0, <25%; 1, 25%-50%; 2, 50%-75%; and 3, >75%). The two values were multiplied together to obtain the immunoreactivity score (IS) of the slice. The staining grade was devided into negative (-, 0 score), weak (+, 1-3 score), moderate (++, 4-6 score) or strong (+++, 7-9 score). If there was a discrepancy in the score, it was regraded until a uniform conclusion was gained.

### Reagents and cell cultures

Rabbit anti-RUFY3 (ab89147) was purchased from Abcam (Cambridge, UK). Rabbit E-cadherin (20874-1-AP), N-cadherin (22018-1-AP), Vimentin (10366-1-AP), Slug(13099-1-AP) and anti-human GAPDH (10494-1-AP) were purchased from Proteintech (Wuhan, China). Human lung cancer cell lines A549, H1299, H1975 and normal bronchial epithelial cells BEAS-2B were cultured using DMEM (Invitrogen, Carlsbad, CA, USA) with 10% fetal bovine serum (Invitrogen) in a humidified atmosphere of 5% CO_2_ incubator.

### Transfection and RNA interference

Cells were transfected in vitro using Lipofectamine 2000(Invitrogen) and following the manufacturer's instructions. The chemically synthesized RUFY3-siRNA and negative control siRNA were purchased from GeneChem Company (Shanghai, China). The sequence of siRNA is as follows: RUFY3-siRNA sequences: 5'-TCTCAAGCATGAACTTGCCTTTAAG-3'. Control siRNA sequences: 5'-UUCUCCGAACGUGUCACGUTT-3'. Transfection efficiency of H1299 and H1975 cells were monitored by western blot.

### Cell invasion assay

Using H1299 and H1975 cell lines, Trans-well Chambers were used to perform cell invasion tests on 24-well plates (0.8-μm pore size; Corning, Costar) coated with ECM gel (40 μL; Sigma), according to the manufacturer's instructions. Cells (2-6×10^4^) were plated in 200μL RPMI DMEM with 0.1% fetal bovine serum in the upper chamber. The lower chamber was filled with 700μL DMEM medium containing 30% fetal bovine serum. After culture for 24 to 72 h, the uninvaded cells were removed using a cotton swab. The invaded cells under the membrane were first fixed with 4% formalin, and then stained with 0.1% crystal violet for microscopic observation and counting. The invaded cells under the membrane were first fixed with 4% formalin, and then stained with 0.1% crystal violet for microscopic observation and counting. The cells were photographed and counted in 10 different fields (×40 magnification).

### Western blot assay

Tissue or cell extracts were treated with a buffer (50 mM Tris-HCl, pH 7.4, 150 mM NaCl, 1% Nonidet P-40, 0.25% Na-deoxycholate, 1 mM EDTA and protease inhibitor cocktail) containing RIPA, Supernatant was collected by centrifugation. Denaturation of an equivalent amount of protein in the SDS buffer at a concentration of 8%, 10% polyacrylamide gel was used according to the molecular weight of the target protein. The separated protein was transferred to the PVDF membrane.The membrane was blocked with 5% non-fat milk TBST (TBS containing 0.05%Tween 20), incubated overnight with the primary antibody, and then incubated with a horseradish peroxidase-conjugated secondary antibody. All PVDF membranes were detected by ECL chemiluminescence (Thermo Fisher Scientific, USA).

### Statistical analysis

SPSS software (version 23.0; SPSS for Windows, Chicago, IL, USA) was used for all statistical analysis. Differences in RUFY3 expression level in lung adenocarcinoma and adjacent normal tissue were quantified using a paired t-test. Rufy3 expression and clinicopathological characteristics were investigated using Mann Whitney test or Kruskal Wallis test. Kaplan-Meier method was used to perform univariate survival analysis. Using the log-rank test, differences in survival rates were determined. The independent predictors of survival were detected using the multivariate Cox regression model. Statistical significance was set at *P*<0.05. Data was expressed as mean ± standard deviation (SD). Student's t test or one-way ANOVA was used to examine the difference between groups.

## Results

### Expression of RUFY3 in lung adenocarcinoma

**Figure 1:**
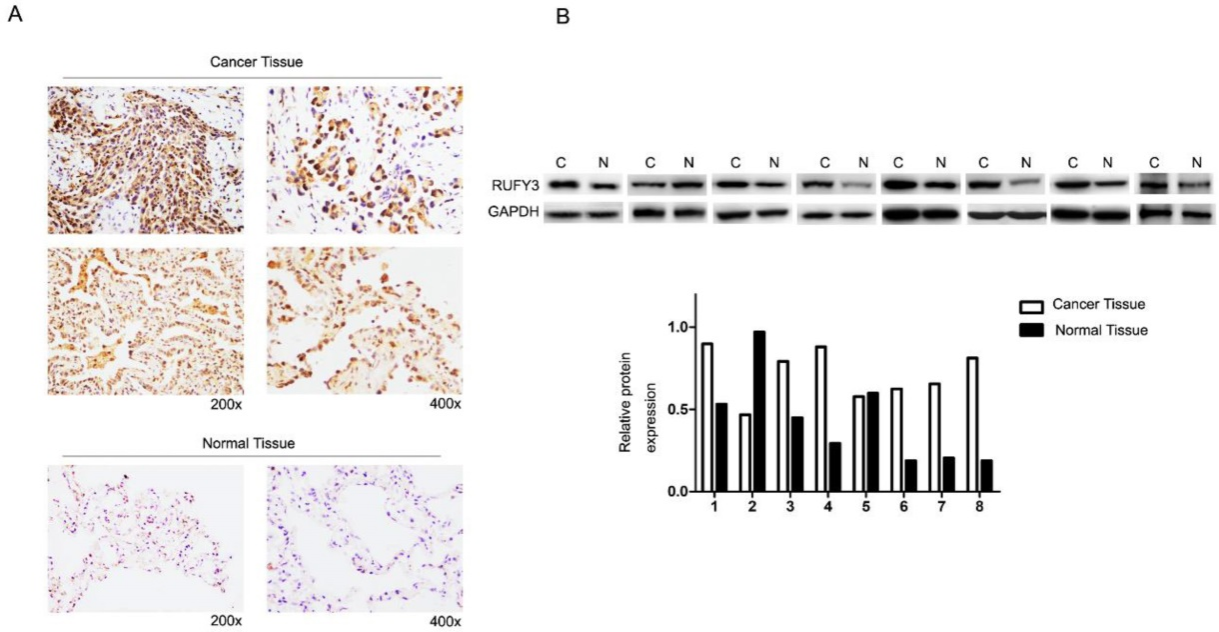
** RUFY3 expression in lung adenocarcinoma** (A) Immunohistochemical assay of RUFY3 expression in 125 freshly collected lung adenocarcinoma biopsies comparing with the normal adjacent tissue at different magnification. (B) Relative protein expression levels were quantified by comparing the gray level of each band using ECL chemiluminescence.

In the 125 lung cancer specimens and adjacent lung tissue, using the immunohistochemical assay we can see that RUFY3 mainly appeared in the cytoplasm in either cancer or normal tissues. As shown in Fig. 1A, the level of RUFY3 expression in lung cancer tissues was significantly higher than in adjacent normal tissue (IS, 6.12 ± 3.58 versus 4.71 ± 3.06, respectively, P = 0.007). These data showed that RUFY3 expression was up-regulated in lung adenocarcinoma tissues. Western blot was also used to verify the conclusion of immunohistochemistry. RUFY3 expression is up-regulated in the vast majority of detected tumor samples compared with the para-carcinoma non-cancerous tissues from the same patient (Fig. 1B). In addition, we used Western blot to detect the expression of RUFY3 in different lung adenocarcinoma cell lines. As shown in Fig. 3A the expression of RUFY3 in lung adenocarcinoma cell lines was higher than that in normal tracheal epithelial cells.

### Correlations between RUFY3 levels and clinicopathological characteristics in lung adenocarcinoma patients

According to immunoreactivity score (IS) of RUFY3, 125 lung adenocarcinoma patients were divided into two groups. Fig. 1 shows RUFY3 expression in different tissues. The expression level of RUFY3 in lung adenocarcinoma was significantly higher than that in normal tissues. Table 1 shows correlations between clinicopathological characteristics and RUFY3 expression levels in patients with lung adenocarcinoma. High cytoplasmic RUFY3 levels were associated with lymph node metastasis, TNM staging, and survival status. However, no significant association between sex, age, smoking, tumor size, differentiation, and RUFY3 expression was observed.

### RUFY3 high expression indicates poor prognosis in lung adenocarcinoma patients

**Figure 2:**
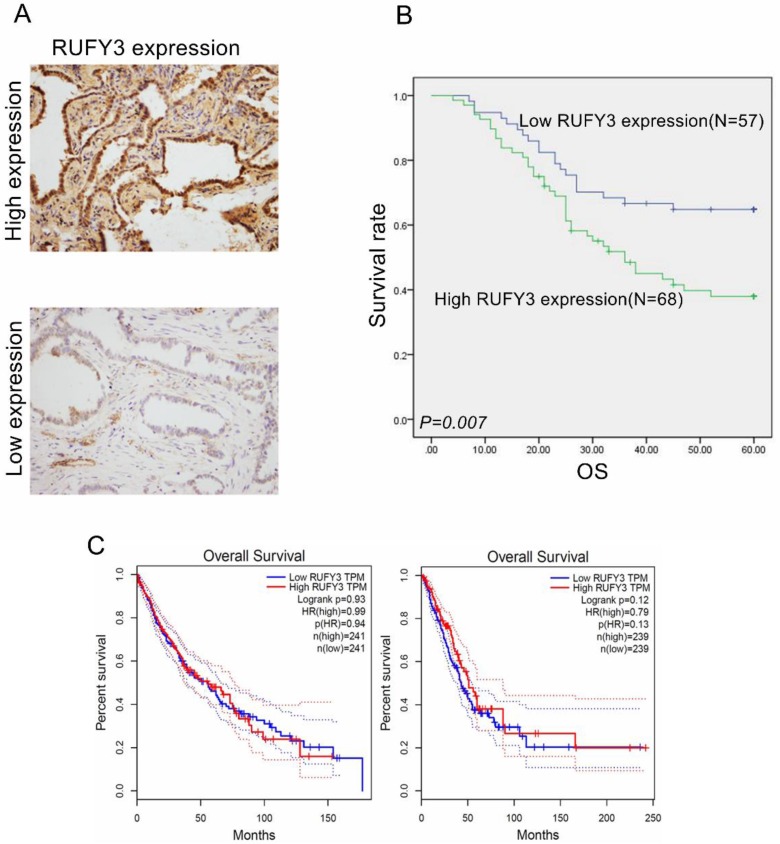
** RUFY3 overexpression heralded poor prognosis of lung adenocarcinoma patients and the survival analysis in other database** (A) IHC staining of high expression (up) and low expression (down) RUFY3 in lung adenocarcinoma tissue (×200); (B) Kaplan-Meier curves indicating the OS rate in 125 patients with lung adenocarcinoma according to the expression status of RUFY3. High level of RUFY3 protein expression correlated with significantly poor OS. (C) The survival analysis of lung cancer prognosis about RUFY3 expression in GEPIA database.

All patients were divided into two groups according to mean expression level to investigate the importance of RUFY3 overexpression in predicting clinical outcomes of lung adenocarcinoma patients. Fig. 2 shows that patients with a high expression level of RUFY3 had shorter survival time than patients with lower expression of RUFY3 (Log-rank test, *P*=0.007). The single Cox regression shows that RUFY3 expression, tumor size, lymph node metastasis, and TNM stage were all risk factors for patient prognosis. Multiple Cox regression analysis showed that high expression level of RUFY3 (*P*=0.044) and tumor size (*P*=0.014) were independent prognostic factors of lung adenocarcinoma (Table 2).

### RUFY3 may affect the migration and invasion of lung cancer cells by mediating EMT

**Figure 3:**
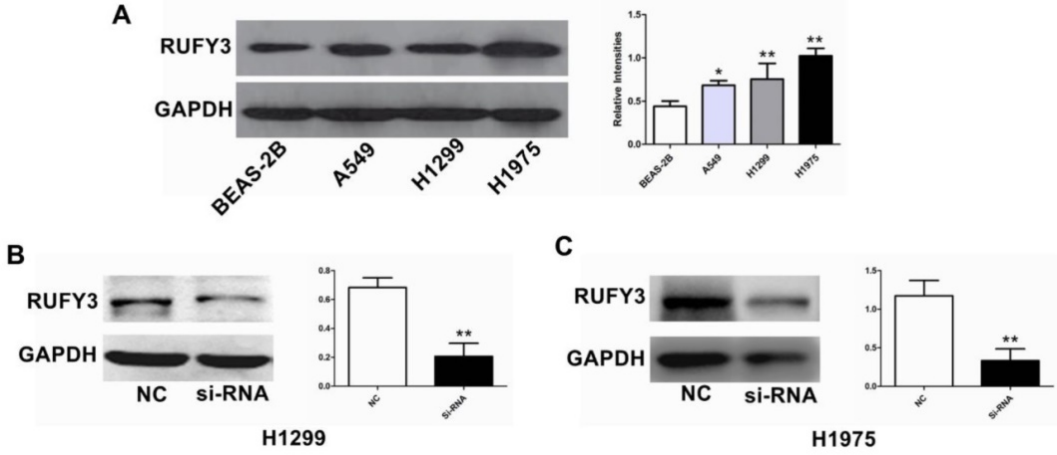
** Comparison of RUFY3 in different cell lines and suppression of RUFY3 by siRNA.** (A): Protein level of RUFY3 in 3 different cancer cell lines (A549, H1299, H1975) and one normal bronchial epithelial cell line (BEAS-2B). (B): Representative data of immunoblotting for RUFY3 in the cells transfected with control siRNA or RUFY3-specific siRNA. (* *P* < 0.05; ** *P* < 0.01).

**Figure 4:**
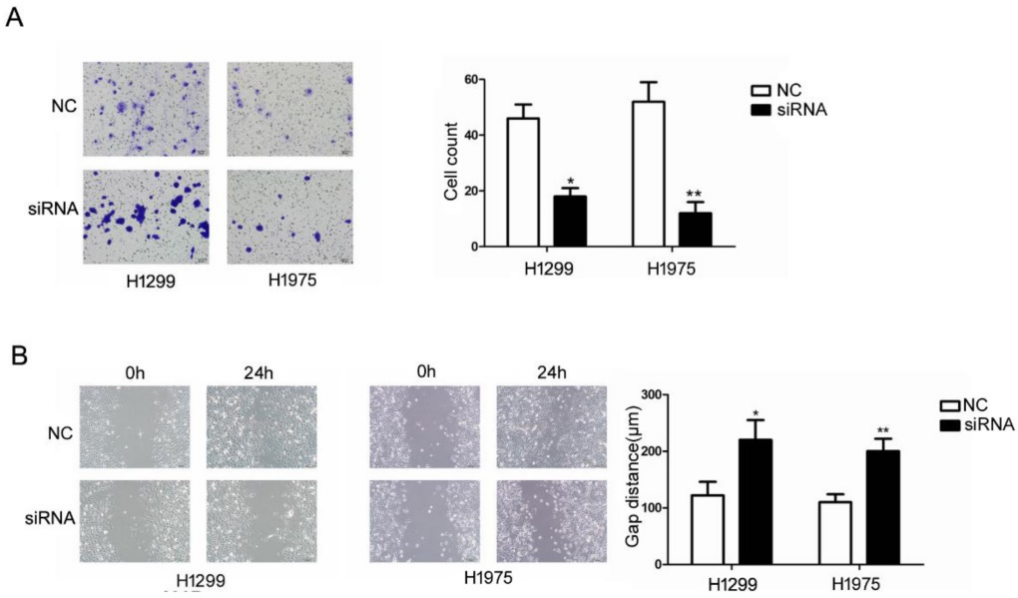
** Suppression of RUFY3 by siRNA and its effect on cell invasion and migration.** Using Trans-well assay to assess the ability of cell migration between si-RNA and control group. (A): representative image data of cells migrated through the Trans-well membrane and quantitative presentation of counted cell number from three separate experiments. (B): Wound healing assay was used to assess the ability of cell migration in the two cell lines of lung cancer, H1299 and H1975, as described in the methods. (* *P* < 0.05; ** *P* < 0.01).

We examined the influence of RUFY3 on the biological behavior of lung cancer cells in vitro. H1299 and H1975 cell lines were divided into RUFY3 silencing group and normal group and were subjected to a wound-healing assay to monitor cell migration. Western blotting was used to analyze the efficacy of RUFY3 RNAi in 2 cell lines, and GAPDH was used as endogenous reference protein (Fig. 3B). We found that no matter in H1299 or H1975 cells, RUFY3 silenced cells showed significantly less of wound-healing cell migration than those in the control group (Fig. 4B). Therefore, these data suggest that RUFY3 can increase the migration ability of lung adenocarcinoma cells. Cell migration is a key step in tumor invasion and metastasis. Therefore, we further investigated whether silencing RUFY3 would affect the invasion ability of these cells. The trans-well chamber was used to quantify the invasion ability of H1299 cells (or H1975 cells) in the RUFY3 silencing group and the control group. Obviously, RUFY3 silencing group had significantly fewer cells crossing the membrane than the control group in both cell lines (Fig. 4A). In summary, these result showed that RUFY3 can affect the migration and invasion of lung adenocarcinoma cells.

**Figure 5:**
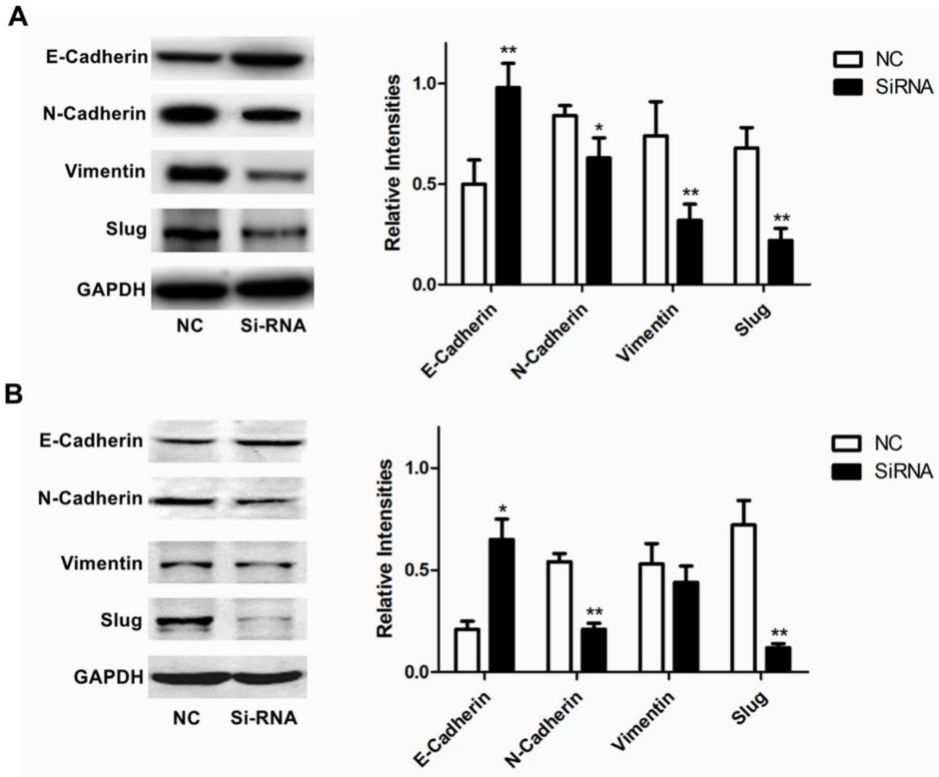
** Role of RUFY3 in regulating proteins associated with EMT.** Specific siRNA targeting RUFY3 and non-specific control siRNA were transfected into the lung cancer cell lines, H1299 (A) and H1975 (B) including representative image data and quantitative presentation(*P< 0.05; **P< 0.01 compared to the cells of control). Total protein was extracted and subjected for immunoblotting of E-cadherin, N-cadherin, Vimentin and Slug as described in the methods. GAPDH was used as loading control.

Furthermore, we analyzed the expression of RUFY3 and EMT markers (E-cadherin, N-cadherin, Vimentin and Slug) in 2 lung adenocarcinoma cell lines after RUFY3 silencing by Western blot. As showed in Fig. 5, we found that after silencing RUFY3, N-cadherin, Vimentin, Slug expression was decreased. Especially Slug, the expression was decreased obviously in both two cell lines. But E-cadherin expression were increased, in H1299 cell line, the change of E-cadherin expression is more obvious (*P*<0.01).

## Discussion

RUFY3 was first identified in Wang's research by a yeast two-hybrid assay and P21-activated kinase-1 (PAK1) as a bait protein. RUFY3 contains an FYVE (Fab1, YOTB/ZK632.12, Vac1 and EEA1) domain in its C terminal. Some previous studies indicated that proteins containing this domain usually play a role in cell polarity, membrane trafficking 7, 20, cytoskeleton remodeling, and some other signal transduction^6^, ^21^. The overexpression of RUFY1, which also belongs to the RUFY family, has led to the persistence of alpha-v integrin in the presynaptic edge.

However, inhibiting the expression of RUFY1 results in a decrease in the expression of alpha-v integrin in peripheral cells. Accordingly, RUFY1 can regulate the transport of integrin in the process of cell migration and tumor invasion 22. In the following study, Wang investigated PAK1 and RUFY3's role in gastric cancer and showed that PAK1 can regulate migration and invasion of gastric cancer mediated by RUFY3 18. RUFY3 can interact with F-actin, myosin, and integrin. Some other studies have also shown that RUFY3 plays various roles in different malignant tumors. Xie's team demonstrated that RUFY3 had a high expression level in colorectal cancer, and could promote cell proliferation, migration, and invasion 23. Another study about colorectal cancer conducted by the same team showed that RUFY3 can interact with FOCK1 and play a role in invasion and metastasis 19.

In the present study, we first used immunochemistry assay to identify expression of RUFY3 in human lung adenocarcinoma and adjacent normal tissues. Positive expression rate in cancer tissues is much higher than in normal tissues (*P*<0.05). The location of RUFY3 was mainly in the cytoplasm. These results were in line with the expression of RUFY3 in colorectal cancers 19. In order to further determine the results, we also use another antibody to repeat the experiment and we got the same result (Fig. 1A, the second line of images of the cancer tissue). These results show that RUFY3 may play an oncogene role in lung adenocarcinoma. As mentioned above, it can interact with the Rab protein family, and there are sufficient evidence that the Rab family plays a role in lung cancer and may indicate poor prognosis 24-26. Concerning the expression level of RUFY3 and clinical characteristics of lung cancer, we discovered that higher expression of RUFY3 may also be associated with lymph node metastasis and TNM staging. In patients with lymph node metastasis positive expression rate of RUFY3 (41/63, 65.1 %) was much higher than in those without lymph node metastasis (27/62, 43.5%). But there was no statistical difference about the tumor size (*P*=0.962), However, there was statistical difference in the expression of RUFY3 in different TNM staging tissues (*P*<0.01). From this we can conclude that RUFY3 mainly play roles in the lymph node metastasis rather than promoting tumor growth. And also there was no distant metastasis data in our study, we will collect the data in further research.

Several recent studies have shown that RUFY3 was correlated with poor prognosis 19, 23. In this study, we also investigated its role in the prognosis of lung adenocarcinoma. According to Kaplan Meier survival analysis, there was a negative association between RUFY3 expression and OS of lung adenocarcinoma patients. Positive RUFY3 expression patients had poorer OS. Single Cox regression showed four clinical characteristics and RUFY3 expression was correlated with patient prognosis. However, after adjusting tumor size, lymph node metastasis, and TNM staging, multiple Cox regression revealed that RUFY3 expression could be considered an independent factor for predicting survival in lung adenocarcinoma patients.

We have showed that higher expression of RUFY3 was associated with lymph node metastasis and TNM staging. Therefore, In the following experiments, we explored the relevant mechanisms. EMT is a key factor in tumor cell invasion, which improves the migration, invasion, anti-apoptosis and extracellular matrix degradation of cancer cells. Here, we showed that inhibiting the expression of RUFY3 in lung adenocarcinoma cells has an effect on the expression of key molecules in the EMT pathway. Consistent with the results of invasion assay, inhibition of RFUY3 expression in the two selected cell lines will lead to decreased expression of N-cadherin, Vimentin and Slug, but will lead to increased expression of E-cadherin. This was also consistent with Xie's study in colorectal cancer 19.

We find that the conclusion in the database is not completely consistent with the conclusion in our research (Fig. 2C), and we think there are several reasons for this result: 1. in this research, we used immunohistochemistry and Western blot to detect the protein expression by using antibodies, while in most database mRNA is more commonly be exanimated. 2. Maybe due to the small sample size of our experiment, in future we will expand the sample size and try to get more accurate results.

In summary, our study was first to identify RUFY3 expression pattern and investigated its relationship with clinicopathological characteristics of lung adenocarcinoma patients. We showed that RUFY3 expression was higher in tumor tissues and that high expression level was bad for the patient prognosis. And the following assays demonstrate that it may be involved in the EMT process. Our results suggest that RUFY3 may act as a novel therapeutic target in lung adenocarcinoma.

## Figures and Tables

**Table 1:** Correlation of RUFY3 expression and pathological characteristics

	n	RUFY3				*P*
		-	+	++	+++	
Total	125	57	19	27	22	
Gender						0.506^#^
Male	54	24	6	13	11	
Female	71	33	13	14	11	
Age						0.177^#^
<60	62	13	21	10	18	
≥60	63	7	18	19	19	
Smoking						0.736^#^
Negative	56	11	16	12	17	
Positive	69	9	23	17	20	
Differentiation						0.590*
Poor	8	3	2	1	2	
Moderate	59	23	11	16	9	
Well	58	31	6	10	11	
Tumor site						0.962*
Right upper lobe	36	16	6	8	6	
Right middle lobe	8	4	1	1	2	
Right lower lobe	28	13	2	8	5	
Left upper lobe	37	18	7	6	6	
Left lower lobe	16	6	3	4	3	
Tumor size						0.564^#^
<3(cm)	61	27	8	14	12	
≥3(cm)	64	30	11	13	10	
Lymph node metastasis						0.019^#^
Negative	62	35	9	9	9	
Positive	63	22	10	18	13	
TNM stage						<0.001*
I	59	37	8	8	5	
II	32	13	5	9	5	
III	34	7	6	9	12	

*^#^P* value was estimated by Mann Whitney test *P value was estimated by Kruskal Wallis test

**Table 2:** Univariate and multivariate regression analysis on risk factors

	Univariate			Multivariate		
	HR	95%CI	*P*	HR	95%CI	*P*
RUFY3 expression	2.046	1.194-3.506	**0.007**	2.246	1.262-3.995	**0.006**
Sex	1.037	0.625-1.720	0.889			
Age	1.073	0.646-1.780	0.786			
Smoking	1.180	0.711-1.959	0.522			
Differentiation	0.580	0.219-1.532	0.152			
Tumor size	1.821	1.191-3.409	**0.009**	1.995	1.175-3.389	**0.011**
Tumor site	0.661	0.274-1.594	0.344			
Lymph node Metastasis	2.015	1.137-3.231	**0.015**	1.866	1.010-3.450	**0.047**
TNM stage	1.613	1.165-2.213	**0.044**	2.176	1.041-4.547	**0.039**
